# Congenital partial pericardial defect confirmed based on spontaneous pneumothorax: A case report and literature review

**DOI:** 10.1016/j.ijscr.2020.09.085

**Published:** 2020-09-15

**Authors:** Naoki Date, Teruya Komatsu, Takuji Fujinaga

**Affiliations:** Department of General Thoracic Surgery, Nagara Medical Center, Gifu, Japan

**Keywords:** Pericardial defect, Pneumothorax, Video-assisted thoracoscopic surgery, Case report

## Abstract

**Introduction:**

Congenital pericardial defects are rare but can cause fatal complications. Most cases are asymptomatic and incidentally detected during a thoracic surgery or autopsy. We report a case of a partial pericardial defect confirmed based on spontaneous pneumothorax.

**Presentation of case:**

A 16-year-old boy with left spontaneous pneumothorax showed pneumopericardium on chest X-ray. Chest computed tomography revealed a partial pericardial defect. Video-assisted thoracoscopic surgery was performed, and a small pericardial defect was confirmed at the level of the upper pulmonary hilum. We did not reconstruct the defect because of the improbability of cardiac herniation.

**Discussion:**

Pneumopericardium combined with pneumothorax suggests the existence of a pericardial foramen. Partial pericardial defects could cause cardiac herniation or strangulation, and pneumothorax may worsen the protruding of the heart. Video-assisted thoracic surgery is an effective method to prevent the recurrence of pneumothorax and determine whether reconstruction of the defect is required.

**Conclusion:**

Video-assisted thoracic surgery should be performed in the case of a pericardial defect combined with pneumothorax.

## Highlights

• Congenital partial pericardial defects are rare but can cause cardiac herniation.
• Pneumopericardium combined with pneumothorax suggests pericardial defect.
• Pneumothorax may worsen the heart protruding through the pericardial defect.
• VATS should be considered in the patients with a pericardial defect.

## 1 Introduction

Congenital pericardial defects are rare and range from partial to complete absence of the pericardium. The frequency is 0.044% of the patients who undergo cardiovascular surgery [1]. Although most cases are asymptomatic and incidentally detected during a thoracic surgery or autopsy, some manifest as chest pain or palpitations. There is a potential risk of a cardiac herniation causing sudden death, particularly in cases of partial absence of the pericardium, so it is crucial to assess the size and location of the defect [2]. Herein, we report a case of a partial pericardial defect detected based on spontaneous pneumothorax and describe the management of the defect.

This case is reported in line with the SCARE criteria [3].

## 2 Presentation of case

**Fig. 1:**
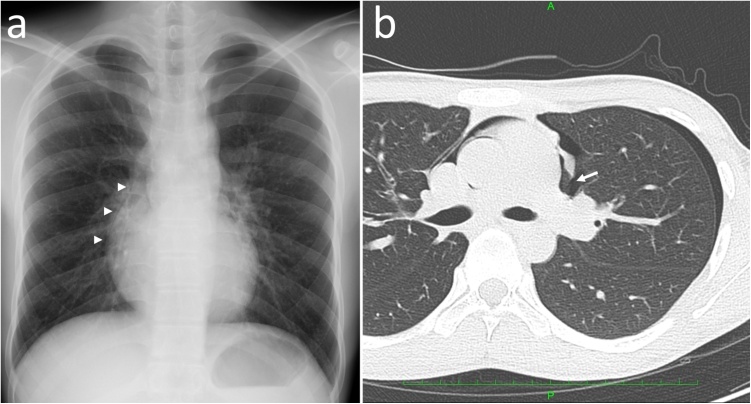
(a) The chest X-ray scan shows left pneumothorax and abnormal air density along the right border of the cardiac shadow (arrow heads). (b) The chest computed tomography scan reveals left pneumothorax, pneumopericardium, and a partial defect of the left pericardium (arrow).

**Fig. 2:**
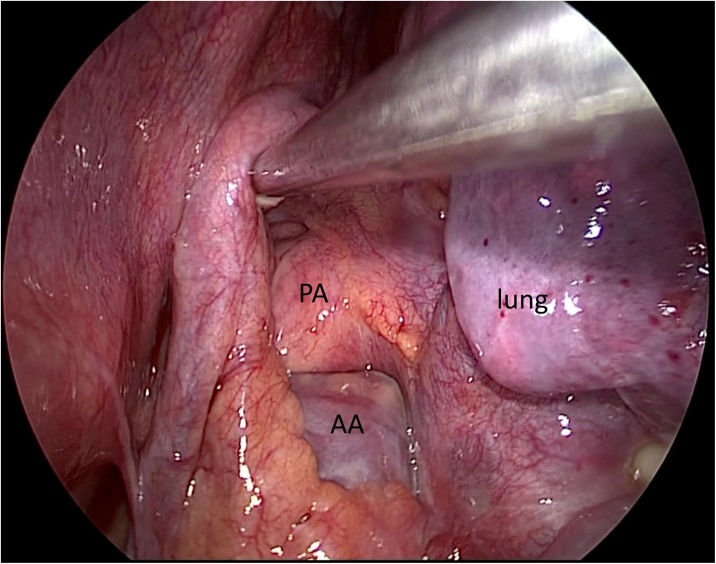
The intraoperative thoracoscopic image shows a partial defect of the left pericardium at the level of the upper hilum. PA: pulmonary artery; AA: atrial appendage.

A 16-year-old boy with no relevant medical history was admitted to our hospital for the surgical treatment of left-sided spontaneous pneumothorax diagnosed at a local hospital. He had chest pain and dyspnea for 1 week. Chest X-ray showed mild pneumothorax in the left lung and abnormal air around the cardiac shadow (Fig. 1a). A 12-lead electrocardiogram showed right-axis deviation within the normal range for his age and a slim body. Chest computed tomography (CT) revealed the left pneumothorax, a small bulla in the lung apex, the pneumopericardium, and a discontinuity of the left pericardium (Fig. 1b). Video-assisted thoracic surgery (VATS) was performed, and the bulla was resected with a linear cutting stapler. An oval defect of the pericardium sized 2 × 4 cm was confirmed at the levels of the left main pulmonary artery and left upper pulmonary vein during the operation. The left atrial appendage was directly observed (Fig. 2). The left phrenic nerve ran along the anteromedial rim of the defect. We did not reconstruct the defect because of the improbability of appendage strangulation or ventricular herniation. The postoperative course was uneventful, and the patient was discharged 2 days later. At the 1-year follow-up, no findings other than slight intercostal neuralgia were observed, and echocardiography was unremarkable.

## 3 Discussion

A congenital pericardial defect is considered to result from failure of the pleuropericardial membranes to close the pleuropericardial foramen by the end of the 5th week of embryonic life [4]. It shows male predominance and mostly occurs on the left side. The partial absent type accounts for 24%–63% of all cases of confirmed pericardial defects [2,5,6]. Symptomatic patients typically complain of chest pain, dyspnea, and palpitation. Approximately one-third of the patients have associated cardiac anomalies, such as an atrial septal defect, mitral stenosis, patent ductus arteriosus, and tetralogy of Fallot [5,6]. In the present case, echocardiography revealed no cardiac abnormalities. Cardiac diseases should be excluded in patients with a congenital pericardial defect.

Most patients with a congenital pericardial defect are suspected or diagnosed using chest X-ray or echocardiography and confirmed using cardiac CT or cardiac magnetic resonance imaging (MRI). Lateral displacement of the heart is a typical finding in patients with complete absence of the pericardium. Chest X-ray may show abnormal presence of the lung tissue between the aorta and the pulmonary artery or between the heart and the diaphragm [2,5,6]. CT or MRI should be considered for patients with a suspected or diagnosed pericardial defect to assess the size and location of the defect [7,8]. Artificial pneumothorax is a histological diagnostic method, which is rarely used nowadays because of its invasiveness and morbidity [2,5]. Pneumopericardium following pneumothorax can prove the existence of a pericardial foramen, as shown in the present case.

The main purpose of surgery for a pericardial defect is to prevent cardiac herniation and strangulation. Patients with complete or large absence of the pericardium, which allows the heart to move back smoothly into the cardiac space, usually need no interventional treatment and show favorable prognosis [6]. Small-to-moderate-sized defects should be considered for surgical treatment because of the possibility of ventricle or atrial appendage strangulation [2,5]. Bennett KR et al. reviewed 44 reported cases, including five fatal cases, of partial pericardial defects [2]. All fatal cases had no symptoms before the fatal attack, and left ventricle strangulation caused sudden death. They suggested that the surgical treatment is warranted even in asymptomatic patients if the defect circumscribes the left ventricle, particularly the body or apex. Patients with severe symptoms should also be considered for surgery. Gatzoulis MA et al. reported ten cases of pericardial defects, four of which included surgical reconstruction [6]. All surgical cases showed improved symptoms postoperatively. The size and location of the defect and presence of symptoms are factors associated with the indication of surgery. In the present case, we did not repair the pericardial defect considering that the defect was limited to the upper region of the heart, and the patient had no history of chest pain before developing the pneumothorax.

Pneumothorax in patients with a pericardial defect can cause cardiac deviation. There are two case reports of cardiac herniation through a large pericardial defect after pneumothorax [9,10]. In cases of a small defect, protruding of the atrial appendage or ventricle could be worsened by pneumothorax, which may lead to strangulation. VATS is an effective measure to prevent recurrence of pneumothorax and determine whether the defect requires repair or not. Therapeutic intervention can be conducted simultaneously with VATS if necessary [5,11].

Enlargement of the defect through pericardiectomy is a traditional treatment method for pericardial defects to prevent cardiac entrapment in the chest cavity. If a patient with a complete or large pericardial defect has severe symptoms, reconstruction of the pericardium could improve symptoms through immobilization of the heart [6]. In cases of a small defect, direct or patch closure has an advantage in preserving the heart's barrier to infections [2]. Amputation of the left atrial appendage is occasionally performed for preventing appendage herniation [12,13]. During surgery, surgeons should be careful not to injure the phrenic nerve. The phrenic nerve usually runs along the anteromedial rim of the defect but may be found behind or even split in the ventral and dorsal segments [2].

## 4 Conclusions

Pneumopericardium combined with pneumothorax suggests the presence of a pericardial defect. A partial pericardial defect could cause fatal complications and require appropriate treatment. VATS should be performed to prevent recurrence of pneumothorax and observe the size and location of the defect.

## Declaration of Competing Interest

The authors report no declarations of interest.

## Sources of funding

Not applicable.

## Ethical approval

An ethics approval committee was not required as this is a case report.

## Consent

Written informed consent was obtained from the patient for the publication of this case report and any accompanying images.

## Author contribution

ND, TK, and TF are the surgeons who operated and treated the patients. The manuscript was drafted by ND. TK and TF supervised the preparation of this report. All authors have read and approved the final manuscript.

## Registration of research studies

NA.

## Guarantor

Naoki Date, Takuji Fujinaga.

## Provenance and peer review

Not commissioned, externally peer-reviewed.
